# 

**Published:** 1883-07

**Authors:** 


### CONTENTS:

Art. I.—A Consideration of Some of the Causes which give Rise to Dental Decay. By Alfred Carpenter, M. D .......97
Art. II.—The Use and Abuse of Amalgam. By A. H. Thompson, D. D. S.......113
Art. III.—Nicotine and its Action upon the Teeth. By David Hepburn.......122
Art. IV.—Dental Caries. By W. D. Miller.......126
Art. V.—What is Vitality of a Tooth. By Dr. Charles Mayr.......130
Southern Dental Associations, Annual Meeting for 1883.......134
Pittsburgh Dental Association.......136

### EDITORIAL, ETC.

The Association Meetings for 1883.......137
Infidelity Among Physicians.......138
Efficacy of Vaccinations.......139
Journalistic Change.......139

### OBITUARY.

Dr. Warren Welch.......140

### MONTHLY SUMMARY.

Inebriety of the Teeth.......141
To Stop Hiccough.......143
To Disguise the Oder of Iodoform.......143
How to Keep Cool.......144
Gluten.......144

